# Remnant cholesterol levels are associated with severity and death in COVID-19 patients

**DOI:** 10.1038/s41598-022-21177-5

**Published:** 2022-10-20

**Authors:** Bibiana Fabre, Nahuel Fernandez Machulsky, Carolina Olano, Darío Jacobsen, María Eugenia Gómez, Beatriz Perazzi, Valeria Zago, Damián Zopatti, Andrés Ferrero, Laura Schreier, Gabriela Berg

**Affiliations:** 1 Departamento de Bioquímica Clínica, Facultad de Farmacia y Bioquímica, Universidad de Buenos Aires, Cátedra de Bioquímica Clínica I, Junín 956 (C1113AAD), Buenos Aires, Argentina; 2 Facultad de Farmacia y Bioquímica, Instituto de Fisiopatología y Bioquímica Clínica (INFIBIOC), Universidad de Buenos Aires, Buenos Aires, Argentina; 3 Facultad de Farmacia y Bioquímica, Consejo Nacional de Investigaciones Científicas y Técnicas (CONICET), Universidad de Buenos Aires, Buenos Aires, Argentina; 4 Facultad de Medicina, Dirección de Estadística y Archivo Médico, Hospital de Clínicas José de San Martin, Universidad de Buenos Aires, Buenos Aires, Argentina; 5 Facultad de Medicina, Director Adjunto Hospital de Clínicas José de San Martin, Universidad de Buenos Aires, Buenos Aires, Argentina

**Keywords:** Biochemistry, Lipids

## Abstract

Coronavirus disease-19 (COVID-19) patients with severe complications present comorbidities like cardiovascular-disease, hypertension and type-2 diabetes mellitus (DM), sharing metabolic alterations like insulin resistance (IR) and dyslipidemia. Our objective was to evaluate the association among different components of the lipid-lipoprotein profile, such as remnant lipoprotein (RLP)-cholesterol, in patients with COVID-19, and to analyze their associations with the severity of the disease and death. We studied 193 patients (68 (29–96) years; 49.7% male) hospitalized for COVID-19 and 200 controls (46 (18–79) years; 52.5% male). Lipoprotein profile, glucose and procalcitonin were assessed. Patients presented higher glucose, TG, TG/HDL-cholesterol and RLP-cholesterol levels, but lower total, LDL, HDL and no-HDL-cholesterol levels (p < 0.001). When a binary logistic regression was performed, age, non-HDL-cholesterol, and RLP-cholesterol were associated with death (p = 0.005). As the COVID-19 condition worsened, according to procalcitonin tertiles, a decrease in all the cholesterol fractions (p < 0.03) was observed with no differences in TG, while levels of RLP-cholesterol and TG/HDL-cholesterol increased (p < 0.001). Lower levels of all the cholesterol fractions were related with the presence and severity of COVID-19, except for RLP-cholesterol levels and TG/HDL-cholesterol index. These alterations indicate a lipid metabolic disorder, characteristic of IR states in COVID-19 patients. RLP-cholesterol levels predicted severity and death in these patients.

## Introduction

Severe acute respiratory syndrome coronavirus 2 (SARS-CoV-2), is a pandemic coronavirus that has quickly spread worldwide since December 2019 being responsible for more than 500 million cases and 6 million deaths globally. Although nearly 80% of the COVID-19 infected patients are asymptomatic or manifest very mild symptoms^1^, the remaining 20% experience severe disease, mainly acute respiratory distress syndrome (ARDS), multiorgan failure and finally death^2^. These complications have been associated with a dysregulated immune response, manifested by lymphopenia and a proinflammatory cytokine storm^3^. Most of the patients suffering the severe pathology present an underlying metabolic dysfunction, characterized mainly by insulin-resistance (IR), type 2 diabetes and higher risk of poor outcomes^4–6^.

Closely linked to IR states and type 2 diabetes, dyslipidemia is very common. The atherogenic dyslipidemia, usually characterized by high triglycerides (TG) and decreased high density lipoproteins (HDL)-cholesterol levels, increases morbidity and mortality. The higher cardiovascular risk in these situations is partially related to elevated plasma concentrations of triglyceride-rich lipoproteins (TRL)-cholesterol, being hepatic overproduction of very low density lipoproteins (VLDL)^7^, the main determinant of plasma triglyceride concentration in IR patients. It has been demonstrated that remnant lipoproteins (RLP)-cholesterol is responsible for a higher increase of C-reactive protein level, as a marker of chronic inflammation, than LDL-cholesterol^8^, highlighting the role of RLPs in the inflammation and the atherogenic process. RLP-cholesterol reflects the cholesterol content of TRL, composed of VLDL and intermediate-density lipoproteins in the fasting state and of these 2 lipoproteins together with chylomicron remnants in the nonfasting state.

Since the SARS-CoV-2 pandemic, several studies have evaluated dyslipidemia in COVID-19 patients. Some studies have described a gradual decrease in total, HDL and LDL-cholesterol across the severity of the infection^9–11^, although others have reported that both low and high LDL-cholesterol levels were significantly associated with higher risk of severe COVID-19^12^. Besides, an increase in TG/HDL-cholesterol ratio has been observed, proposing the use of this ratio as a biochemical marker of COVID-19 severe prognosis^13^. However, no data have been reported neither about RLP-cholesterol in these patients nor regarding the behavior of these lipoproteins according to the severity of the infection.

Given the scarce knowledge about SARS-CoV-2 and metabolic alterations up-to-date, our aim was to study blood lipid profiles in patients with COVID-19, especially to disclose the association of RLP-cholesterol and TG/HDL-cholesterol as surrogate markers of IR with COVID-19 severity. Besides, this manuscript attends to add data to the scientific community and to improve the understanding of this infectious disease.

## Methods and patients

This is a retrospective study from the Hospital de Clínicas, José de San Martin, University of Buenos Aires, between March 1st 2020 and April 30th 2021. Data from a total of 193 consecutive patients (68 (29–96) years; 49.7% male) with a definitive diagnosis of SARS-CoV-2 infection confirmed through positive reverse transcriptase polymerase chain reaction (RT-PCR) were obtained. Exclusion criteria were age < 18 years old, pregnant women, lack of clinical or biochemical data for this study, and death in the first 24 h after admission. The baseline clinical data, ongoing treatment, laboratory tests and clinical outcomes were retrospectively collected from the electronic medical records. Data of previous pathologies and therapy before admission, as well as during the hospitalization, were also recorded. The admission at Intensive Care Unit (ICU), hospital infection and death data were also recorded. The death of the patients occurred in all cases in the hospital, and the average time of hospitalization of these patients was 20 (9–69) days. In parallel, a control group included ambulatory subjects, COVID-19 negative, who attended consecutively for a routine check-up, at the same Hospital during the same period of time, (Controls, 46 (18–79) years; 52.5% male). According to the Institution’s protocols, the work was carried out following the guidelines of the Declaration of Helsinki of the World Medical Association.

Blood samples were obtained after overnight fasting; for COVID-19 patients in the first 24 h during hospitalization, according to medical requests; in the case of negative COVID-19 patients, when attending a scheduled appointment to the Laboratory. A blood routine examination analyzer (Sysmex XN-1000) was used for routine blood tests. The neutrophil (N) count, lymphocyte (L) count, red cell volume distribution width (RBW) were recorded, the N/L ratio (NLR) was calculated. Total-cholesterol, TG and glucose were measured in serum, using commercial kits (Roche Diagnostics) in a Cobas C-501 autoanalyzer, mean coefficients of variation (CV) values for these parameters were < 1.9% for intra-assay CVs and < 2.4% for interassay CVs. HDL-cholesterol and LDL-cholesterol were determined by standardized homogeneous assays (Roche Diagnostics), intra assay CV < 2.0% and inter-assay CV < 2.6%. Non HDL-cholesterol was calculated as Total-cholesterol minus HDL-cholesterol, and RLP-cholesterol as Total-cholesterol minus LDL-cholesterol and HDL-cholesterol.

Procalcitonin was measured only in patients diagnosed with COVID-19 by a chemiluminescent method on the Centaur XPT autoanalyzer, Siemens. The detection limit and functional sensitivity reported by the manufacturer is 0.04 ng/ml and < 0.05 ng/ml, respectively.

The Hospital Ethic Committee (Comité de Etica del Hospital de Clínicas de la Universidad de Buenos Aires, HHS USA No OHRP00012655) approved the study protocol (2020-0870070/07-20). Informed consent was waived by the Hospital Ethic Committee (Comité de Etica del Hospital de Clínicas de la Universidad de Buenos Aires, HHS USA No OHRP00012655), given the retrospective and observational nature of the study.

### Statistical analyses

The distribution of variables was tested (kurtosis and skewness) and the results were expressed as mean ± standard deviation (SD) or median (range), accordingly. Pearson for parametric distribution data, or Spearman test for non-parametric distribution data were used for correlation analysis. T-test or Mann–Whitney test were used for mean or median differences comparison, and Kruskal Wallis analysis was performed to evaluate differences between medians when more than two groups were analyzed. We then used a binary logistic regression analysis to test whether metabolic and lipidic profile were predictors of COVID-19 and death, controlling for necessary confounders as age. Prior to the onset of the study, the number of patients required for detecting differences of at least one standard deviation, with an statistical power of 80% and an α = 0.05, were determined. According to this, for the studied parameters the number of cases should not be less than 173. All the analyses were conducted with SPSS 23.0 statistical software.

## Results

The sociodemographic, clinical and biochemical characteristics of the study population are shown in Table 1. Among the COVID-19 patients 21% presented diabetes mellitus (DM) (n = 41); 16% high blood pressure (n = 31) and 6% renal chronic disease (n = 11). The control group presented 21.5% of DM patients (n = 43) and 10% presented high blood pressure (n = 20). Diabetic or hypertensive patients and controls were under the appropriate treatment.

**Table 1:** Sociodemographic, clinical and biochemical characteristics of the study population.

	Controls (n = 200)	Patients (n = 193)	p value
Age (years)	46 (18–79)	68 (29–96)	< 0.001
Sex (male, %)	105 (52.5)	96 (49.7)	0.328
Glucose (mg/dl)	101 (76–537)	133 (68–522)	< 0.001
**Comorbidities, n (%)**			
DM	43 (21.5)	41 (21.2)	0.902
High blood pressure	20 (10)	31 (16)	0.210
Chronic renal disease	–	11 (6)	–
RDW	12.9 (11.4–28.4)	13.7 (11.5–28.8)	< 0.001
NLR	1.79 (0.57–14.07)	8.06 (0.75–139)	< 0.001
Triglycerides (mg/dl)	125 (39–1282)	150 (44–637)	< 0.001
Total-cholesterol (mg/dl)	186 (98–338)	140 (58–274)	< 0.001
LDL-cholesterol (mg/dl)	113 (25–250)	74 (8–194)	< 0.001
HDL-cholesterol (mg/dl)	48 (18–89)	33 (5–67)	< 0.001
Non HDL-cholesterol (mg/dl)	133 (32–275)	105 (32–237)	< 0.001
TG/HDL-cholesterol	2.49 (0.57–71.22)	4.55 (1.13–84.8)	< 0.001
RLP-cholesterol (mg/dl)	18.5 (1–112)	27.0 (8.8–85)	< 0.001

Results expressed as Median (range) or n (percentage) as appropriate. Mann–Whitney and Chi square test.

*DM* diabetes mellitus, *RDW* red cell volume distribution width, *NLR* neutrophil-to-lymphocyte ratio, *TG* triglyceride, *RLP* remnant lipoprotein.

The table shows significant differences in age, glucose levels, RDW, NLR and the lipid-lipoprotein profile between groups. Besides, COVID-19 patients presented lower levels of total Cholesterol, LDL-cholesterol, HDL-cholesterol, non-HDL-cholesterol and higher levels of RDW, NLR, TG, TG/HDL-cholesterol and RLP-cholesterol than controls.

We performed a binary logistic regression introducing the dichotomous variable (0 = control, 1 = COVID-19) as the dependent variable and TG, TG/HDL-cholesterol, non-HDL-cholesterol and RLP-cholesterol as independent variables. We found that all these variables were associated with COVID-19, showing the highest association with TG/HDL-cholesterol (p = 0.001, OR 1.315 [CI 1.21–1.543]), and RLP-cholesterol (p < 0.0001, OR 1.108 [CI 1.061–1.157]), even after adjusting by age.

In COVID-19 patients RLP-cholesterol positively correlated with NLR (r = 0.250, p = 0.001) and RDW (r = 0.140, p = 0.05).

When analyzing COVID-19 population between those who died or not as consequence of SARS-CoV-2, we observed that those patients who died were older and presented higher procalcitonin levels, RDW and NLR. Regarding the lipoprotein profile, in patients who died lower levels of total-cholesterol, LDL-cholesterol and non-HDL-cholesterol were observed, however RLP-cholesterol levels were higher (Table 2). On the other hand, when we performed a binary logistic regression introducing the dependent variable (0 = not dead, 1 = dead) and age, HDL-cholesterol, LDL-cholesterol, TG/HDL-cholesterol, RDW, NLR and RLP-cholesterol as independent variables. We found that age (p = 0.002, OR:1.054 [IC:1.019–1.091]), RDW (p = 0.005, OR 1.267 [IC:1.073–1.497]), NLR (p = 0.001, OR 1.106 [IC: 1.058–1.156]) and RLP-cholesterol (p = 0.010, OR 1.051 [IC:1.012–1.092]) were associated with death, even after adjusting for comorbidities such as diabetes, hypertension and chronic renal failure.

**Table 2:** Clinical and biochemical characteristics of COVID-19 patients according to death.

Variables	Survived (n = 129)	Dead (n = 64)	p
Age (years)	67 (29–87)	73 (31–96)	< 0.0001
Sex (male, %)	60 (47)	35 (55)	0.328
Glucose (mg/dl)	128 (74–522)	139 (68–309)	0.277
DM (n, %)	25 (19)	14 (22)	0.451
RDW	13.4 (11.5–23.5)	14.4 (12.4–28.8)	< 0.0001
NLR	5.77 (0.75–46.14)	14.4 (0.79–139.43)	< 0.0001
Triglycerides (mg/dl)	151 (52–637)	143 (44–424)	0.989
Procalcitonin (ng/ml)	0.11 (0.01–52.51)	0.44 (0.01–85.4)	< 0.0001
Total-cholesterol (mg/dl)	150 (58–274)	132 (63–200)	0.019
LDL-cholesterol (mg/dl)	81 (8–194)	62 (10–152)	< 0.0001
HDL-cholesterol (mg/dl)	33 (9–67)	32 (5–56)	0.492
Non HDL-cholesterol (mg/dl)	110 (36–237)	97 (32–179)	0.021
TG/HDL-cholesterol	4.5 (1.3–33.7)	4.6 (1.1–84.8)	0.327
RLP-cholesterol (mg/dl)	18.5 (10–112)	27 (8.8–88)	< 0.001

Results expressed as Median (range). Mann–Whitney or Chi square test according to data distribution.

*DM* diabetes mellitus, *RDW* red cell volume distribution width, *NLR* neutrophil-to-lymphocyte ratio, *TG* triglyceride, *RL*P remnant lipoprotein.

Among those patients who died (n = 64), 50 of them (78%) were hospitalized in the ICU.

Once again, we evaluated which variables were associated with death through a binary logistic regression, finding a strong association between death and ICU hospitalization, even after adjusting for age (p = 0.006, OR 9.456 [IC: 1.934–46.231]).

Finally, we divided COVID-19 patients into tertiles according to procalcitonin concentration (1st tertile 0.010–0.070 ng/ml, 2nd tertile 0.071–0.39 ng/ml, 3rd tertile > 0.390 ng/ml) (Table 3). As the levels of procalcitonin increased, the number of death patients was higher; besides, lower levels of all the lipoproteins cholesterol were observed, except from those of RLP-cholesterol and the TG/HDL-cholesterol ratio.

**Table 3:** Clinical and biochemical characteristics of COVID-19 patients according to procalcitonin tertiles.

	Procalcitonin tertiles			p value
	0.010–0.070 ng/ml (n = 46)	0.071–0.390 ng/ml (n = 60)	> 0.391 ng/ml (n = 54)	
Age (years)	65 (30–96)	68 (29–94)	70 (29–93)	0.151
Sex (male, %)	21 (46)	35 (58)	38 (70)	0.429
Glucose (mg/dl)	123 (78–262)	147 (83–330)	127 (68–309)	0.009
DM (yes, %)	9 (20)	14 (23)	9 (17)	0.579
Triglycerides (mg/dl)	158 (44–509)	143 (63–637)	171 (65–424)	0.169
Total cholesterol (mg/dl)	136 (71–252)	144 (72–250)	123 (58–215)	0.001
LDL-cholesterol (mg/dl)	75 (23–194)	78 (25–164)	55 (8–153)	< 0.001
HDL-cholesterol (mg/dl)	36 (18–61)	35 (12–67)	25 (5–55)	< 0.001
Non HDL-cholesterol (mg/dl)	103 (32–218)	108 (43–237)	92 (36–184)	0.035
TG/HDL-cholesterol	4.3 (1.1–23.1)	4.0 (1.6–30.5)	5.9 (2.3–84.8)	< 0.001
RLP-cholesterol (mg/dl)	24 (9–59)	27 (13–88)	32 (13–85)	< 0.001
Death (n, %)	9 (19)	20 (33)	31 (57)	< 0.001

*DM* diabetes mellitus.

Chi square test. Kruskal Wallis Test.

## Discussion

In this study we observed an increase in TG and a decrease in all the lipoproteins-cholesterol levels in patients with COVID-19, except for an increase in RLP-cholesterol and TG/HDL-cholesterol. Moreover, the increase in remnants levels were not visualized by non-HDL-cholesterol, which was also decreased in SARS-CoV-2 patients.

It has been previously reported that high levels of TG and low levels of HDL-cholesterol are associated with the severity and progression of COVID-19^13,14^. The hypercatabolic status during the acute infection in these patients would justify the low levels of lipoproteins cholesterol observed, however the increased in TG levels is paradoxical. Nevertheless, the TG/HDL-cholesterol index is a surrogate marker of insulin-resistance, associated with obesity and diabetes, both pathologies very prevalent in severe COVID-19.

Besides, it is well known that remnant particles are an independent risk factor for cardiovascular disease as well as for chronic inflammation and all-cause mortality^7,15^. RLP-cholesterol levels are highly associated with low grade of inflammation, even more than LDL-cholesterol, given that remnants can be taken by macrophages in the intima, without the necessity of previous modifications like oxidation or glycation^8^. The role of remnants in the chronic inflammatory process is clear and the presence of high levels of these particles have also been associated with an overall pro-inflammatory and pro-thrombotic state^16^. Ballout et al.^17^ study evaluated the particle numbers and sizes by NMR spectroscopy. They revealed that patients admitted to the ICU with COVID-19 presented a high number of TRL  particles, and particularly the very small and small subfractions. In our study, we calculated the RLP-cholesterol levels from Total-cholesterol minus LDL-cholesterol and HDL-cholesterol and we observed that this cholesterol subfraction was associated with severity and death in COVID-19 patients. Moreover, our study shows a direct association of RLP-cholesterol with RDW as well as with NLR. These hematological parameters have recently been proposed as novel inflammatory markers for the diagnosis and prognosis of COVID-19 infection^18^.

Hypertriglyceridemia during hospitalization has been associated with mortality in patients with COVID-19, even after adjusting for multiple factors such as age, gender, obesity among many other factors^19^. Inflammation and infection have previously associated with hypertriglyceridemic states, attributed to a lower catabolism of RLP triglycerides as consequence of lower activity of lipoprotein lipase^19^. Our results would be in coincidence with this results; given that RLP is a remnant from TRL, its persistence in circulation could be consequence of decreased lipoprotein lipase activity.

Given that previous studies proposed that procalcitonin may be a marker of severity in COVID-19, and that serial procalcitonin measurement can be used in predicting the prognosis^20,21^, we divided COVID-19 patients according to procalcitonin tertiles. We verified that those patients with the highest procalcitonin values showed the lower Cholesterol levels in all the lipoproteins subfractions except in RLP levels. RLP-cholesterol concentration was higher as well as the TG/HDL-cholesterol ratio in those patients. Besides, the higher proportion of death was observed in this group.

The decrease in non-HDL-cholesterol in COVID-19 is an important result to highlight. This cholesterol fraction, which includes LDL and RLP cholesterols, did not show the increase in the remnant subfractions, given the contribution of LDL-cholesterol to non-HDL value. In this regard, the measurement of remnants, as a part of the lipoprotein profile, would be an additional metabolic marker for COVID-19 patients.

Our study has some limitations. We were unable to obtain the body mass index data from patients and controls. Considering the importance of obesity on the lipoprotein profile as well as on COVID-19, adjusting our results by body mass index would have been an interesting and clarifying possibility, which would improve the analysis of the results. Another limitations was the lack of hs-C Reactive Protein data, however, we have included RDW and NLR, which have arisen as good alternatives for the study of the inflammatory process. Besides, the number of patients limits the possibility to obtain more robust conclusions.

## Conclusion

In this study we demonstrate for the first time that RLP-cholesterol and non-HDL-cholesterol are associated with death in COVID-19. More studies are necessary to prove that these parameters can be used as markers in the prognostic and/or risk-stratification in patients with COVID-19. The measurement of these cholesterol fractions is an alternative that can be easily performed in clinical laboratories.

## Data Availability

The datasets used and/or analyzed during the current study available from the corresponding author on reasonable request.
